# Efficacy and Safety of *Lactobacillus plantarum* K50 on Lipids in Koreans With Obesity: A Randomized, Double-Blind Controlled Clinical Trial

**DOI:** 10.3389/fendo.2021.790046

**Published:** 2022-01-19

**Authors:** Minji Sohn, Ga Yoon Na, Jaeryang Chu, Hyunchae Joung, Byung-Kook Kim, Soo Lim

**Affiliations:** ^1^ Department of Internal Medicine, Seoul National University Bundang Hospital, Seoul National University College of Medicine, Seongnam, South Korea; ^2^ Microbiome Research Laboratory, Chong Kun Dang BiO Corporation (CKD BiO Corp.) Research Institute, Ansan, South Korea; ^3^ Head of Probiotics & Microbiome Part, Chong Kun Dang Bio Corporation (CKD BiO Corp.) Research Institute, Ansan, South Korea

**Keywords:** *Lactobacillus plantarum*, probiotics, obesity, body fat, lipid

## Abstract

**Background:**

Only few studies have investigated the role of probiotics in the development of obesity. We aimed to determine the efficacy and safety of an intake of *Lactobacillus plantarum* K50 (*LPK*) on body fat and lipid profiles in people with obesity.

**Methods:**

This randomized, double-blind, placebo-controlled, clinical trial involved 81 adults with a body mass index of 25–30 kg/m^2^ who were assigned randomly to a diet including 4 × 10^9^ colony-forming unit of *LPK* or a placebo. Changes in body fat, anthropometric parameters, and biomarkers of obesity were compared using a linear mixed-effect model.

**Results:**

After 12 weeks of treatment, body weight, fat mass, and abdominal fat area did not change significantly in the two groups. However, total cholesterol levels decreased from 209.4 ± 34.4 mg/dL to 203.5 ± 30.9 mg/dL in the *LPK* group, but increased from 194.7 ± 37.5 mg/dL to 199.9 ± 30.7 mg/dL in the placebo group (P = 0.037). Similarly, triglyceride levels decreased from 135.4 ± 115.8 mg/dL to 114.5 ± 65.9 mg/dL in the *LPK* group, with a significant difference between groups. *LPK* supplementation also tended to decrease leptin levels compared with placebo. It also changed the distribution of gut microbiota significantly, with an increase in *L. plantarum* and a decrease in *Actinobacteria*, both of whose changes in abundance were correlated with changes in visceral adiposity, with borderline significance.

**Conclusion:**

A 12-week consumption of *LPK* reduced the total cholesterol and triglyceride levels significantly with favorable alterations in microbiota, suggesting potential benefits for controlling blood lipid profiles.

## Introduction

The prevalence of obesity has been increasing worldwide (1). Obesity in Asia is increasing rapidly with rapid environmental changes. Asians have a genetically distinct body composition and are adapted to a high carbohydrate nutritional intake (2). Now, Western-type food intake coupled with reduced physical activity and an urban lifestyle are leading to an increase in obesity in this region (3). South Korea is not an exception to this trend (4). The rates of noncommunicable diseases in Korea, such as coronary heart disease, hypertension, and type 2 diabetes are increasing almost linearly along with increased average body mass index (BMI) (4). Indeed, obesity is associated with a multitude of comorbidities spread across several different organs (5). Comorbidities can be classified into three major domains: the metabolic domain includes cardiovascular diseases and type 2 diabetes; the mechanical domain includes asthma, chronic back pain, and knee osteoarthritis; and the mental domain includes depression and anxiety (6–8).

Of note, dysbiosis of the gut microbiota is now recognized as a major contributor to chronic human diseases including obesity (9, 10). More specifically, people with obesity have larger numbers of *Firmicutes* and fewer *Bacteroidetes* than lean people (9). *Firmicutes* and *Bacteroidetes* are associated with the development of obesity, thereby affecting the host’s acquisition of nutrients and energy balance (10, 11). In a study with 68 obese subjects and 47 controls, *Bifidobacterium animalis* tended to be associated with normal weight whereas *Lactobacillus (L.) reuteri* was associated significantly with obesity (12).

From a different perspective, a meta-analysis of 15 randomized controlled trials has found that administration of probiotics, mostly containing 10^9^ to 10^11^ colony-forming units (CFU) of *L.* and *Bifidobacterium* species, resulted in a significant reduction in body weight and BMI, compared with placebo (13). A recent proof-of-concept study showed that administration of pasteurized *Akkermansia muciniphila* for 3 months decreased plasma lipopolysaccharide in people with obesity and metabolic impairment (14). Thus, changes in the intestinal microflora have emerged as indicators of obesity and metabolic regulation, indicating that their alteration can be both a cause and treatment target for obesity and metabolic disorders (15).

*L.* species have been studied widely for their metabolic benefits, such as reducing body fat mass, body weight, and cholesterol levels (13). A significant weight loss of 1.5% (P < 0.0001) was achieved after 6 months of supplementation with *L.* and *Bifidobacterium* in adults with obesity or overweight status (16). In a study of healthy Japanese subjects, body fat mass decreased significantly after the administration of *L. gasseri* SBT2055 for 12 weeks (17). In one study, there was no significant decrease in the body weight of subjects after the ingestion of soymilk fortified with *L. planetarium* A7 for 8 weeks (18), and another found no significant decrease in body fat after taking *L. gasseri* BNR17 for 12 weeks (19). In our previous study, administration of 5 × 10^9^ CFU of *L. sakei* resulted in a mean body weight reduction of 2 kg with borderline significance (20).

Thus, the anti-obesogenic effects of probiotics are inconclusive in humans. However, treatment with an *L. plantarum* K50 (*LPK*), which is isolated from Kimchi, was reported to decrease weight in mice (21). Therefore, the aim of this study was to examine whether the administration of probiotics containing *LPK* would have beneficial effects on body fat, body weight, and related metabolic factors in people with obesity.

## Materials and Methods

### Subjects and Study Design

We enrolled healthy men and women aged 20 to 65 years with a BMI of 25–30 kg/m^2^ who understood the content of the study and agreed to participate in this clinical trial. Individuals were excluded if they reported any of the following criteria: had continuously taken drugs that affect weight, lipid, blood glucose metabolism, immune and inflammatory reactions within one month prior to screening; had continuously consumed foods that could affect intestinal health within 1 month before screening; had uncontrolled hypertension or diabetes mellitus requiring treatment; had abnormal liver function with increased aspartate aminotransferase (AST), or alanine aminotransferase (ALT) levels more than 3 times the upper limit of normal; or abnormal kidney function (serum creatinine levels > 1.4 mg/dL); had thyroid dysfunction; had participated in a commercial antiobesity program or had been treated with a calorie-restricted diet within 3 months; had a weight change of 5% or more within 3 months; or had undertaken surgery such as gastroplasty or enterectomy for weight loss.

**Figure 1:**
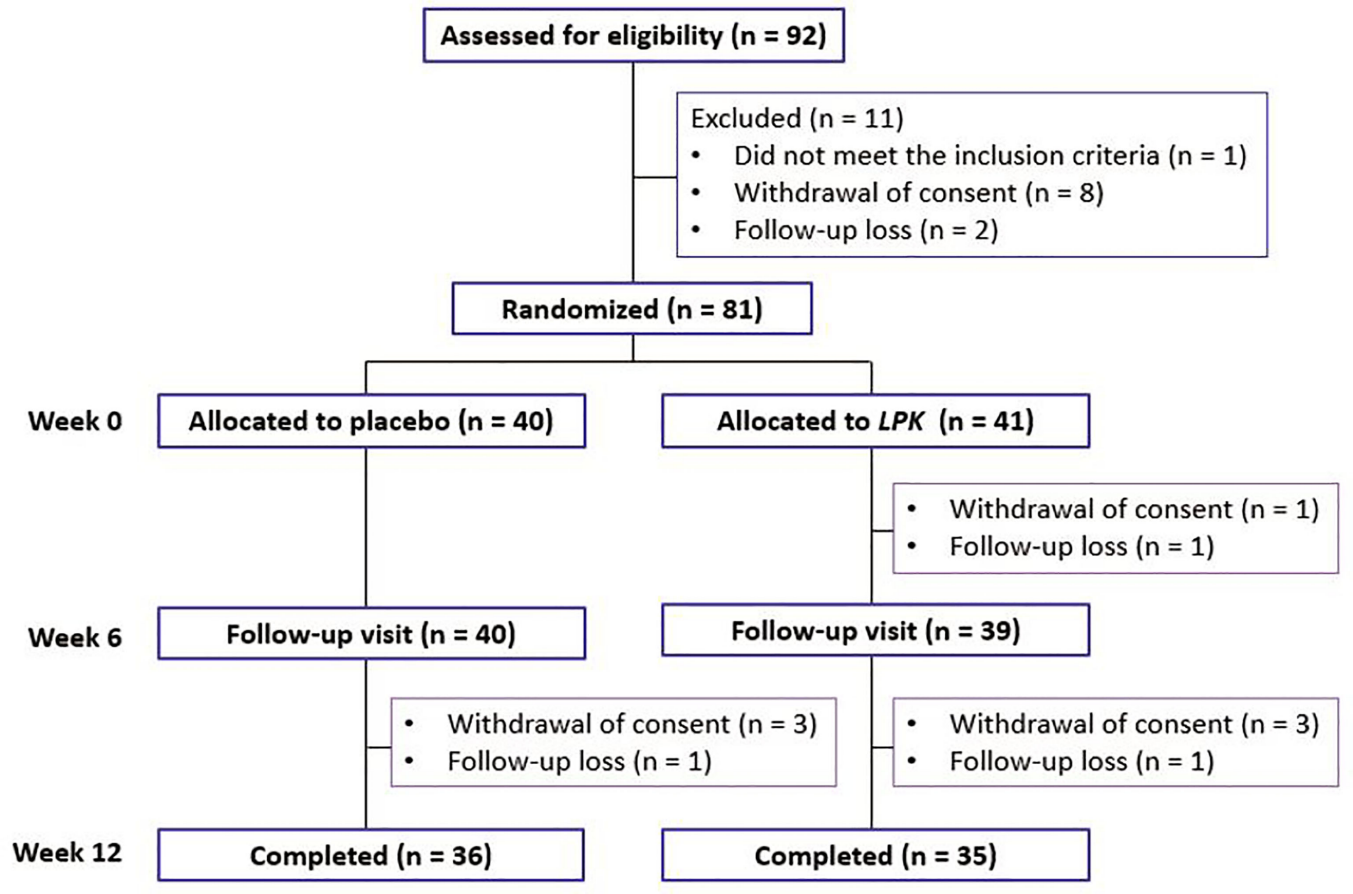
Distribution of the study participants during the study period. LPK, *Lactobacillus plantarum* K50.

In all, 92 patients were screened for this study; 81 were eligible to participate and were randomized to either the *LPK* or placebo groups (**Figure 1**). Participants received two daily allocations of 2 × 10^9^ CFU of *LPK* (total 4 × 10^9^ CFU/day) or the equivalent placebo for 12 weeks (**Supplementary Figure S1**). The participants were instructed by a trained research coordinator to follow the standard instructions for a healthy lifestyle from the run-in period to the end of the study. They were advised to exercise regularly at least three times per week for ≥30 min for each session. The nutritional instructions were developed based on the Dietary Reference Intakes for Koreans (KDRIs) published by the Ministry of Health and Welfare and The Korean Nutrition Society (http://kns.or.kr/English/Publication.asp). Detailed information about these instructions is provided in the **Supplementary Material**. Good compliance with the treatment was defined as taking over 80% of the allocation.

During the study period, six participants in the *LPK* group and four in the placebo group dropped out (**Figure 1**). Three did not visit for the follow-up measurement and one did not meet the criteria for medication compliance, and the others withdrew from the study without specific reasons. The overall compliance in both groups was >94%.

This study was conducted according to our management standards with approval of the Ethics Committee of Seoul National University Bundang Hospital (SNUBH; B-1901-516-002). The subjects provided written informed consent after listening to detailed explanations from the researchers. This study was registered at the Clinical Research Information Service of the Republic of Korea (KCT0003944; https://cris.nih.go.kr/cris/en/use_guide/cris_introduce.jsp).

### Study Materials

The *LPK* strain was isolated from Kimchi incubated in a modified MRS medium (21). It was cultured in MRS broth at 37°C for 24 h. After that, it was inoculated into a fermenter with the optimized medium. Fermentation was performed under constant pH (6.0 ± 0.5) and agitation at 37°C for 16 h. After fermentation, the culture medium was removed, and cells were harvested, concentrated, and lyophilized. Lyophilized *LPK* was ground and packaged in polyethylene and aluminum bags. Packaged probiotics were stored at 4°C before dispatch. Placebo and probiotic capsules were provided by CKDBiO Corp. (Ansan, South Korea). Probiotic capsules were composed of *LPK* and microcrystalline cellulose powder, named CKDB156. Microcrystalline capsules with texture, color, and odor identical to those of the probiotic were used as the placebo vehicle. Quality checking of both products including coliform group bacteria, heavy metals, residual pesticides, and nutrients was performed and approved by the Korea Advanced Food Research Institute of the Korea Food Industry Association (Uiwang, South Korea).

### Primary and Secondary Outcomes

The primary outcome of this study was any change in the subjects’ body fat mass from the baseline to 12 weeks after beginning treatment. The key secondary outcomes were changes in BMI, body weight, waist circumference, and abdominal adipose tissue area from baseline to 12 weeks. Other secondary outcomes were changes in metabolic parameters from baseline to 12 weeks.

### Assessment of Body Composition

Body weight and height were measured using standard methods with the subject in light clothing. BMI was calculated as weight (kg) divided by height (in meters) squared. Waist circumference was measured at the umbilical level. Systolic blood pressure (SBP) and diastolic blood pressure (DBP) were measured with an electronic blood pressure meter while the subjects were seated. Blood pressure was measured twice at a 5-min interval, and the mean value was used in the analysis.

To measure whole-body fat mass, muscle mass, and percent body fat in the study participants, dual-energy X-ray absorptiometry (DXA; Horizon W, Hologic Inc., Bedford, MA, USA) was used. Scanning was performed with the subject supine, and all scans were completed within 15 min.

Abdominal adipose tissue areas were quantified by a single scouting view of a computed tomography (CT) scan (Somatom Sensation 16; Siemens, Munich, Germany). Subjects were examined in the supine position with their arms outstretched overhead to decrease beam hardening or streak artifacts. Scanning was performed at 90-kV exposure. The exposure time was 0.1 s, and the scanning time was 0.5 s. A 10-mm CT slice scan was acquired at the umbilical level to measure the total abdominal and visceral fat areas. Adipose tissue attenuation was determined by measuring the mean value of all pixels within the range of –190 to –30 Hounsfield units. The images were converted into files compatible with a commercial software program (Rapidia; 3DMED, Seoul, South Korea). To assess the visceral adipose tissue (VAT) area, each abdominal image was edited by erasing the image exterior to the innermost abdominal wall muscles with a mouse-driven cursor, and the resulting images were saved in separate files.

### Collection of Lifestyle Information and Measurement of Biochemical Parameters

Alcohol consumption, smoking status, and menstruation status were investigated through questionnaires. The eating habits of the participants were investigated using a recommended food score questionnaire.

The subjects fasted for 12 h overnight, and venous blood samples were taken for biochemistry assays. Plasma glucose concentration was measured using the glucose oxidase method (747 Clinical Chemistry Analyzer, Hitachi, Tokyo, Japan). Fasting plasma insulin level was measured using a radioimmunoassay (Linco, St. Charles, MO, USA). AST and ALT (NADH-UV method) and creatinine (Jaffe’s kinetic method) levels were measured using an Architect Ci8200 Analyzer (Abbott Laboratories, Abbott Park, IL, USA). The estimated glomerular filtration rate (eGFR) was calculated using the CKD-EPI creatinine equation (22). Serum high-sensitivity C-reactive protein (hsCRP) level was measured *via* a high-sensitivity automated immunoturbidimetric method (CRP-Latex [II]X2; Denka Seiken Co., Tokyo, Japan). Concentrations of total cholesterol, TG, HDL-C, low-density lipoprotein cholesterol (LDL-C), and free fatty acids were measured using a 747 Clinical Chemistry Analyzer (Hitachi).

Adiponectin levels were measured using an enzyme-linked immunosorbent assay (ELISA; Otsuka Pharmaceutical Co., Tokyo, Japan). Levels of tumor necrosis factor-α (TNF-α) were measured in duplicate in serum samples using a commercial ELISA kit (R&D Systems, Minneapolis, MN, USA, intra-assay coefficient of variation [CV] < 6.2%, inter-assay CV < 11%, detection sensitivity 20 pg/mL). Levels of interleukin (IL)-6 were measured in duplicate in serum samples using the ELISA method (R&D Systems, intra-assay CV < 8.4%, inter-assay CV < 9.6%, detection sensitivity 0.003–0.014 ng/mL). Plasma glucagon concentrations were determined using a validated ELISA (Mercodia AB, Uppsala, Sweden). Serum concentrations of total ketones were measured using enzymatic immunoassay kits (Nittobo Medical Co. Ltd., Tokyo, Japan). Analyses of lipopolysaccharide-binding protein (LBP), resistin, and soluble cluster of differentiation 14 (sCD14) were performed using commercially available ELISA kits (AdipoGen Life Sciences, San Diego, CA, USA) according to the manufacturer’s instructions in a certified laboratory (GCCL, Yongin, South Korea). Assays for leptin were performed in duplicate by ELISA (R&D Systems).

### Microbial Sequencing

Metagenomic DNA extraction, library construction, and sequencing for microbiome analysis were all performed by Macrogen, Inc. (Seoul, South Korea). Fecal samples were collected from subjects within 3 days of the study visit date and stored at –80°C until analyzed. Metagenomic DNA was extracted from the fecal samples using DNeasy PowerSoil Pri kits (QIAGEN, Hilden, Germany) and underwent quality control inspection. Qualified samples were subjected to tagmentation that combines the fragmentation of DNA, and 5′ and 3′ adapter-ligation processes. Adapter-ligated DNA fragments were subjected to polymerase chain reaction amplification of the V3 and V4 regions of 16s rRNA genes and purified by gel electrophoresis. Prepared libraries were loaded into flow cells and amplified to generate clusters. Then, sequencing was performed using the MiSeq system (Illumina Inc., San Diego, CA, USA). Sequencing data were converted to FASTQ files for analysis. These FASTQ data were processed for data trimming and taxonomic classification using the Greengenes reference database (http://greengenes.secondgenome.com).

### Statistical Analysis

Based on a previous study using two strains of *L* (23)., the sample size for this study was calculated as 35 per group; this would give 90% power to detect differences in the abdominal fat area. Considering the relatively high follow-up loss rate in obesity studies in general, we planned to enroll 50 participants in each group, assuming a maximum 30% loss rate. The complete randomized intention-to-treat population of this study was used for evaluating efficacy and safety. Baseline demographics and clinical data are reported for all subjects as the number and (percentage) and the mean ± standard deviation (SD). Comparisons between mean subject values at baseline were analyzed using Student’s t test for continuous variables and chi-square tests for categorical variables. Anthropometric and biochemical parameters at 12 weeks were compared with their baseline values using a linear mixed-effect model with fixed effects of treatment, time, treatment-by-time interaction, and subject-specific random intercepts. We used IBM SPSS Statistics for Windows version 22.0 (IBM Corp., Armonk, NY, USA) and P < 0.05 was considered significant.

For microbiota, relative abundance was used for analysis, including α-diversity calculated using the Shannon index. Gut microbial dissimilarities between groups at the genus level were visualized by principal coordinates analysis (PCoA) and permutational multivariate analysis of variance (PERMANOVA) using Bray–Curtis dissimilarities at the genus level. The linear relationship between the changes in clinical values and relative abundance of microbiota before and after dietary supplementation were subjected to Pearson’s correlation analysis. Paired Student’s t tests or Wilcoxon signed-rank tests were applied to detect mean differences in the gut microbial features at baseline and posttreatment measurements. P-values were adjusted using the Benjamini–Hochberg method for multiple comparisons. The microbiome analysis was conducted using R software version 4.1.0 (R Development Core Team, Vienna, Austria) and RStudio version 1.4.1103 (RStudio, Boston, MA, USA).

## Results

### Baseline Characteristics

**Table 1** lists the baseline characteristics of study participants. The ratio of men to women was similar between the two groups. The mean age was 47.8 ± 11.7 years in the *LPK* group and 45.5 ± 10.0 years in the placebo group. The baseline BMI values were 27.1 ± 1.5 kg/m^2^ in the *LPK* group and 27.3 ± 1.6 kg/m^2^ in the placebo group. Most baseline parameters were not significantly different between the two groups except for the levels of liver enzymes, hsCRP, and TNF-α.

**Table 1:** Baseline characteristics of study participants.

Variables	Placebo (*n*=40)			*LPK* (*n*=41)			P
Age, year	45.5	±	10.0	47.8	±	11.7	0.335
Sex, male/female	16/24			16/25			0.930
Body weight, kg	74.0	±	8.8	74.6	±	9.9	0.752
Body mass index, kg/m^2^	27.3	±	1.6	27.1	±	1.5	0.559
Waist circumference, cm	93.0	±	5.1	93.2	±	5.6	0.886
Systolic blood pressure, mmHg	125.5	±	10.2	124.0	±	10.9	0.548
Diastolic blood pressure, mmHg	78.6	±	9.3	76.7	±	10.1	0.376
Fasting glucose, mg/dL	98.5	±	11.8	97.5	±	10.8	0.698
Total ketone, μmol/L	214.1	±	121.5	244.5	±	164.8	0.404
Free fatty acid, μEq/L	590.6	±	245.4	614.9	±	208.2	0.661
AST, IU/L	32.6	±	9.5	26.0	±	6.5	0.001
ALT, IU/L	41.2	±	22.2	29.2	±	11.6	0.004
Creatinine, mg/dL	0.74	±	0.16	0.72	±	0.14	0.606
eGFR, mL/min/1.7 m^2^	105.1	±	11.4	104.2	±	10.7	0.714
hsCRP, mg/L	0.87	±	0.85	0.48	±	0.31	0.018
TNF-α, pg/mL	3.5	±	1.1	2.9	±	0.8	0.005
Total cholesterol, mg/dL	194.7	±	37.5	209.4	±	34.4	0.070
Triglyceride, mg/dL	119.0	±	44.1	135.4	±	115.8	0.418
HDL-cholesterol, mg/dL	55.9	±	11.2	59.2	±	13.3	0.242
LDL-cholesterol, mg/dL	125.2	±	30.7	129.3	±	24.0	0.505
Alcohol drinker, Yes/No	20/20			26/15			0.223
Smoker, Yes/No	6/34			7/34			0.799
Smoking amount, cigarette/day	13.7	±	11.9	11.8	±	5.6	0.651
Menstruation, Yes/No/NA	14/10/16			14/11/16			0.983
Recommended food score	21.1	±	7.6	20.3	±	8.2	0.658

The data are presented as the mean ± standard deviation or as the number of subjects. Student’s t test for continuous variables and chi-square test for categorical variables were used to compare differences between groups. LPK, Lactobacillus plantarum K50; AST, aspartate aminotransferase; ALT, alanine aminotransferase; eGFR, estimated glomerular filtration rate calculated by CKD-EPI creatinine equation; hsCRP, high-sensitivity C-reactive protein; NA, not applicable.

### Changes in Body Fat Mass and Other Anthropometric and Body Composition Parameters

Total body fat mass—the primary outcome measure of this study— did not change significantly in either group (**Table 2**). BMI, body weight, and abdominal adipose tissue area also did not change in either group. For waist circumference, the *LPK* group showed a nonsignificant tendency to decrease from 93.2 ± 5.6 cm to 91.3 ± 4.9 cm (P = 0.093) (**Table 2**).

**Table 2:** Changes in body composition in the LPK and placebo groups after 12 weeks.

Variables	Placebo (*n*=40)							*LPK* (*n*=41)							P^†^
	Baseline			12 weeks			P^*^	Baseline			12 weeks			P^*^	
Body weight, kg	74.0	±	8.8	74.8	±	9.1	0.208	74.6	±	9.9	74.2	±	10.0	0.726	0.440
BMI, kg/m^2^	27.3	±	1.6	27.5	±	1.9	0.227	27.1	±	1.5	27.0	±	1.7	0.572	0.521
Waist circ., cm	93.0	±	5.1	92.4	±	6.1	0.461	93.2	±	5.6	91.3	±	4.9	0.093	0.495
Fat mass, kg	27.3	±	3.8	27.9	±	4.6	0.892	28.0	±	4.3	27.1	±	3.3	0.257	0.380
Lean mass, kg	43.1	±	8.0	43.6	±	8.1	0.053	43.3	±	8.3	42.9	±	8.5	0.567	0.064
Body fat, %	38.2	±	5.6	38.1	±	5.7	0.303	38.5	±	5.7	38.7	±	5.7	0.984	0.434
TAT, cm^2^	379.3	±	95.3	362.4	±	112.6	0.266	373.1	±	75.8	369.2	±	72.8	0.923	0.420
VAT, cm^2^	130.9	±	55.7	127.4	±	55.1	0.686	113.9	±	45.1	111.6	±	43.2	0.977	0.753
SAT, cm^2^	248.4	±	76.2	235.1	±	85.8	0.158	247.5	±	70.1	245.9	±	62.7	0.978	0.334

Presented as the mean ± standard deviation. LPK, Lactobacillus plantarum K50; BMI, body mass index; TAT, total adipose tissue; VAT, visceral adipose tissue; SAT, subcutaneous adipose tissue. *P-values for the differences between the groups for 12 weeks were obtained from a linear mixed-effect model. ^†^P-values for differences within each group were obtained from a linear mixed-effect model.

### Changes in Blood Pressure and Biochemical Parameters

The SBP/DBP and other metabolic parameters and their changes are listed in **Table 3**. Most parameters at baseline were not different between the two groups except for the levels of liver enzymes, hsCRP, and TNF-α. The SBP tended to decrease in the *LPK* group and tended to increase in the placebo group, but these differences were not significant (P = 0.059).

**Table 3:** Changes in blood pressures and biomarkers in the LPK and placebo group after 12 weeks.

Variables	Placebo (*n*=40)								*LPK* (*n*=41)								P^†^
	Baseline				12 weeks			P*	Baseline				12 weeks			P*	
SBP, mmHg	125.5	±	10.2	127.9		±	9.6	0.057	124.0	±	10.9	121.9		±	10.1	0.436	0.059
DBP, mmHg	78.6	±	9.3	77.1		±	8.0	0.247	76.7	±	10.1	74.7		±	7.2	0.285	0.977
Glucose, mg/dL	98.5	±	11.8	97.3		±	7.9	0.824	97.5	±	10.8	94.5		±	6.3	0.154	0.400
Insulin, μIU/mL	9.2	±	4.7	10.0		±	4.9	0.250	6.8	±	3.7	6.7		±	3.1	0.658	0.273
Glucagon, pg/dL	144.2	±	77.6	145.1		±	69.8	0.674	109.6	±	44.2	108.6		±	33.7	0.964	0.783
Total ketone, μmol/L	214.1	±	121.5	185.7		±	80.6	0.109	244.5	±	164.8	210.4		±	117.6	0.358	0.891
Free fatty acid, μEq/L	590.6	±	245.4	578.6		±	152.2	0.780	614.8	±	208.2	647.4		±	206.4	0.475	0.479
Leptin, ng/mL	2.4	±	1.5	3.3		±	2.6	0.175	2.8	±	1.8	2.6		±	1.7	0.383	0.092
Adiponectin, μg/mL	20.0	±	12.0	20.2		±	11.7	0.958	21.4	±	12.3	23.0		±	13.4	0.796	0.914
TNF-α, pg/mL	3.5	±	1.1	3.6		±	0.9	0.911	2.9	±	0.8^‡^	2.8		±	1.0	0.818	0.795
IL-6, pg/mL	2.2	±	4.7	1.5		±	2.5	0.389	0.6	±	0.2	0.7		±	0.4	0.639	0.380
sCD14, ng/mL	1493	±	191	1326		±	312	0.003	1454	±	334	1398		±	356	0.427	0.098
LBP, ng/mL	14304	±	2226	11094		±	1781	<0.001	13699	±	2566	11008		±	1789	<0.001	0.356
Resistin, ng/mL	26.4	±	12.7	26.3		±	12.6	0.277	26.3	±	12.4	26.6		±	14.8	0.725	0.280
AST, IU/L	32.6	±	9.5	29.9		±	7.8	0.178	26.0	±	6.5^‡^	24.6		±	6.1	0.130	0.733
ALT, IU/L	41.2	±	22.2	37.0		±	20.1	0.171	29.2	±	11.6^‡^	24.1		±	10.9	0.001	0.996
Creatinine, mg/dL	0.74	±	0.16	0.70		±	0.16	0.088	0.72	±	0.14	0.68		±	0.13	0.035	0.885
eGFR, mL/min/1.7 m^2^	105.1	±	11.4	108.9		±	9.7	0.068	104.2	±	10.7	108.1		±	10.2	0.04	0.759
hsCRP, mg/L	0.87	±	0.85	0.65		±	0.40	0.122	0.48	±	0.31^‡^	0.46		±	0.25	0.867	0.168
Total cholesterol, mg/dL	194.7	±	37.5	199.9		±	30.7	0.159	209.4	±	34.4	203.5		±	30.9	0.113	0.037
Triglyceride, mg/dL	119.0	±	44.1	143.0		±	73.1	0.009	135.4	±	115.8	114.5		±	65.9	0.784	0.014
HDL-C, mg/dL	55.9	±	11.2	55.4		±	11.4	0.798	59.2	±	13.3	59.6		±	13.3	0.609	0.861
LDL-C, mg/dL	125.2	±	30.7	129.1		±	27.2	0.212	129.3	±	24.0	128.7		±	25.6	0.847	0.268

Data are presented as the mean ± standard deviation. LPK, Lactobacillus plantarum K50; SBP, systolic blood pressure; DBP, diastolic blood pressure; TNF-a, tumor necrosis factor-a; IL-6, interleukin-6; sCD14, soluble cluster of differentiation 14; LBP, lipopolysaccharide-binding protein; AST, aspartate aminotransferase; ALT, alanine aminotransferase; eGFR, estimated glomerular filtration rate calculated by CKD-EPI creatinine equation; hsCRP, high-sensitivity C-reactive protein; HDL-C, high-density lipoprotein cholesterol; LDL-C, low-density lipoprotein cholesterol. *P-values for the differences within each group were obtained from a linear mixed-effect model. ^†^P-values for differences between groups after 12 weeks were obtained from a linear mixed-effect model. ^‡^Significant difference between the two groups at baseline.

The total cholesterol level decreased from 209.4 ± 34.4 mg/dL to 203.5 ± 5.3 mg/dL in the *LPK* group but increased from 194.7 ± 37.5 mg/dL to 199.9 ± 30.7 mg/dL in the placebo group (P = 0.037). Similarly, the TG level decreased from 135.4 ± 115.8 mg/dL to 114.5 ± 65.9 mg/dL in the *LPK* group but increased significantly from 119.0 ± 44.1 mg/dL to 143.0 ± 73.1 mg/dL in the placebo group (P = 0.009), indicating significant differences between the groups (P = 0.014).

Leptin levels decreased from 2.8 ± 1.8 ng/mL to 2.6 ± 1.7 ng/mL in the *LPK* group but increased from 2.4 ± 1.5 ng/mL to 3.3 ± 2.6 ng/mL in the placebo group, showing a marginal difference between the two groups (P = 0.092). Serum creatinine, AST and ALT levels, and other biochemical parameters were not different between the groups (**Table 3**). There was no significant difference between the two groups in terms of changes in other biomarkers such as LBP, resistin, and sCD14.

### Changes in Gut Microbiota

**Figure 2:**
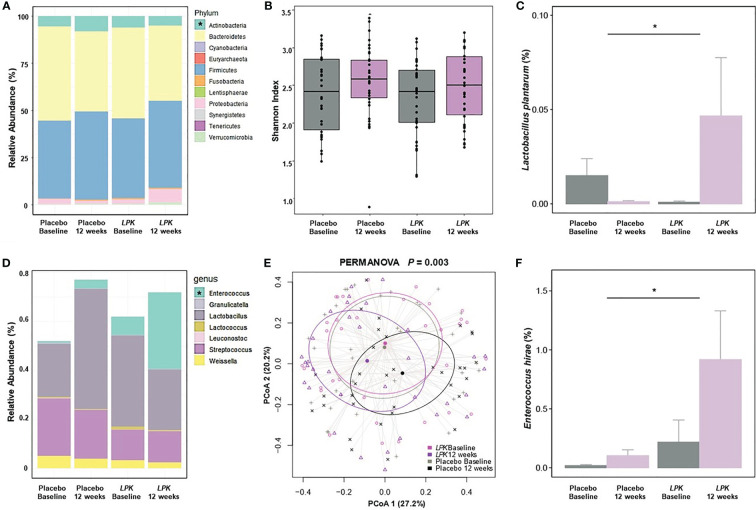
Changes in gut microbiota after a 12-week dietary supplementation with *LPK* or placebo. **(A)** Relative abundance of the phyla. **(B)** The alpha diversity is shown as a Shannon index. **(C, F)** Comparison of logarithmic mean changes of rarefied counts of *Lactobacillus plantarum* and *Enterococcus hirae*. **(D)** Relative abundance of the genera in the order *Lactobacillales*. **(E)** The beta diversity as PCoA of the genus in the order *Lactobacillales. LPK*, *Lactobacillus plantarum* K50; PCoA, principal coordinates analysis. Asterisks show between-group differences for changes after 12-week supplementation. with a Benjamini–Hochberg adjusted P-value < 0.05.

The changes in the gut microbiota are shown in **Figure 2**. At the phylum level, *LPK* supplementation significantly decreased *Actinobacteria* abundance compared with placebo, which was positively correlated with VAT area (*r* = 0.24, P = 0.051) (**Figure 2A**). Overall α- and β-diversity values were not different between the two groups although *LPK* supplementation significantly increased the abundance of *L. plantarum* (0.05% ± 0.18% vs –0.01% ± 0.05%) (P < 0.05) (**Figures 2B, C**). Changes in *L. plantarum* raw counts were inversely correlated with changes in the abdominal adipose tissue area before and after therapy, with borderline significance (*r* = –0.25, P = 0.073, **Supplementary Figure S2**). Among the genera in the order *Lactobacillales*, abundance in *Enterococcus*, which was inversely related to obesity status (fat mass: *r* = –0.41, P = 0.063; body weight: *r* = –0.22, P = 0.070), increased significantly in the *LPK* group compared with the placebo group (0.70% ± 2.32% vs 0.09% ± 0.28%) (P < 0.05) (**Figure 2D**). The relative abundance of the order *Lactobacillales* was similar between the groups. However, the composition of genera changed differently resulting in a significant between-group difference (PERMANOVA = 0.003) (**Figure 2E**). Specifically, the abundance of *Enterococcus hirae* was significantly increased in the *LPK* group (0.70% ± 2.33% vs 0.09% ± 0.28%) compared with the placebo group (P < 0.05) (**Figure 2F**). This might have been caused by *LPK* supplementation because it showed a positive correlation with the abundance of *L. plantarum* (*r* = 0.22, P = 0.047).

### Adverse Events

There were no significant differences between the groups in terms of the occurrence, type, degree of symptoms, and relevance to intervention (**Table 4**). The symptom severities of adverse reactions were all mild, and any relationship with the intervention was not considered relevant. There were no deaths or serious adverse events requiring hospitalization.

**Table 4:** Adverse events.

	Placebo	*LPK*	P
Adverse event	1/2	2/3	1.000
Serious adverse event	0/0	0/0	–
*Type*			
Pruritus	0/0	1/1	1.000
Facial laceration	1/1	0/0	0.494
Low back pain	0/0	1/1	1.000
Insomnia	0/0	1/1	1.000
Vasovagal syncope	1/1	0/0	0.494
*Relevance to intervention*			
Definitely related	0/0	0/0	–
Probably related	0/0	0/0	–
Possible related	0/0	0/0	–
Probably not related	1/2	2/3	1.000
Definitely not related	0/0	0/0	–
Unknown	0/0	0/0	–

Presented as the number of subjects/number of cases. Fisher’s exact test was used to compare the differences in the numbers of subjects between groups. LPK, Lactobacillus plantarum K50.

## Discussion

In this study, the subjects’ body weight, fat mass, and abdominal adipose tissue area did not change significantly after a 12-week administration of *LPK*, resulting in no significant differences compared with the placebo group. However, the total cholesterol level decreased in the *LPK* group and increased in the placebo group, resulting in a significant between-group difference of 11.1 mg/dL. Similarly, the TG level decreased in the *LPK* group and increased in the placebo group, resulting in a significant between-group difference of 44.9 mg/dL.

*L. plantarum* is a versatile lactic acid-producing bacterium found in many fermented foods (24). *L. plantarum* is widely employed in the industrial fermentation and processing of raw foods; it is generally recognized as safe and has received a Qualified Presumption of Safety status from the European Food Safety Authority (25, 26). Oral administration of the live *L. plantarum* strain K21 alleviated high-fat diet-induced obesity in a mouse model (27). In another study with high-fat-fed mice, *L. plantarum* supplementation reduced obesity-induced metabolic abnormalities and adipose tissue inflammation (28). In a study with Indonesian subjects, consumption of indigenous probiotic *L. plantarum* Dad-13 powder in overweight adults decreased the body weight and BMI significantly (29). In a Japanese study, the ingestion of heat-treated *L. plantarum* OLL2712 reduced body fat accumulation, glycemic deterioration, and chronic inflammation in overweight, healthy adults (30). Thus, the effects of *L. plantarum* on altering the metabolic status differ among studies, suggesting that it depends on the study subjects and the strain used (31, 32).

In our study, the administration of 4 × 10^9^ CFU of *LPK* to people with a baseline BMI ≥ 25 kg/m^2^ led to reductions in total cholesterol and TG levels. During a prior clinical trial, twelve weeks of consumption of a mixture of *L. plantarum* strains CECT7527, CECT7528, and CECT7529 decreased total cholesterol levels as observed (33). The cholesterol-lowering efficacy of lactic acid-producing bacteria is reported to be a result of producing short chain fatty acids (SCFAs) (9). Of note, in a study with diet-induced obese mice, treatment with 1 × 10^9^ CFU *LPK* increased the concentrations of SCFAs significantly (21). SCFAs, especially acetate, lowered fat accumulation in metabolic tissues as well as serum TG and total cholesterol levels in an animal study (34). Other SCFAs such as butyrate and propionate protected against diet-induced obesity and regulated gut hormones such as glucagon-like peptide-1, glucose-dependent insulinotropic polypeptide (GIP), peptide YY, and ghrelin (35). GIP regulates TG turnover and promotes TG clearance from the blood by increasing the deposition of fat in adipocytes (36, 37). Based on these findings, the reductions in total cholesterol and TG levels by administration of *LPK* in our study might have been caused by increases in SCFAs and changes in the composition of the gut microbiota.

Dietary supplementation with *LPK* in our study changed the gut microbiota favorably with a decrease in the abundance of *Actinobacteria* and an increase in *Enterococcus*. The decrease in *Actinobacteria* species might be linked with a decrease in obesity, which was increased in obese compared with lean twin subjects (38). Administration of a specific strain of *Bifidobacteria* (M13-4), belonging to the *Actinobacteria*, was reported to be associated with weight gain in a high-fat diet study in rats (39). *Enterococcus* species abundance was reported to be correlated negatively with excessive weight gain and increased leptin (40). We also found that *LPK* supplementation increased the abundance of *Enterococcus hirae* in the order *Lactobacillales* to which *LPK* belongs. In a study of rats fed a high-fat diet, 24-h supplementation of *Enterococcus hirae* (2 mg/10^10^ CFU) decreased total cholesterol and TG levels and alleviated insulin resistance (41). A recent study with hypercholesterolemic rats, administration of specific strains of *Enterococcus faecium* decreased total cholesterol, LDL-C, and TG levels by regulating the genes involved in lipid metabolism, such as *CYP8B1*, *CYP7A1*, *SREBP-1*, *SCD1*, and *LDL-R* (42). Thus, the benefit of *LPK* on lipid profiles might be driven by favorable alterations in the distribution of gut microbiota.

Human gut microbiota can be substantially diverse between individuals and alter over time, changing according to age, genetics, and environment (43). In addition, ethnicity is another important factor affecting the type and abundance in gut microbiota (44, 45). A multi-omics study of 46 East Asian and White participants living in the San Francisco Bay Area revealed marked differences between ethnic groups in bacterial richness and community structure; White individuals were enriched for the mucin-degrading *Akkermansia muciniphila* whereas East Asian subjects had increased levels of multiple bacterial genera including *Blautia, Bacteroides*, and *Streptococcus* (44). In another study with multi-ethnic groups, ethnicity exhibited the largest effect size across participants; notably, the influence of ethnicity on the gut microbiota was retained even after controlling for all demographic, dietary factors, and other covariates (45). In this context, the effects of probiotics, even originating from the same strain, may be different according to the subject’s ethnicity.

Food consumption alters the gut microbiome profile in humans and such dietary patterns are important for the link between gut microbiota and body composition changes (46–48). Conversely, changes in the composition and activity of the gut microbiota might affect body weight and its composition (49, 50). However, as previous studies have reported (14, 51), the intake of probiotics intervention did not change the overall gut microbiotic diversity in the present study. Therefore, instead of showing *all* microbiota, only the changes in specific microbiota at the genus or species level that seem to be clinically meaningful are presented. In this study, the abundances of *L. plantarum* and *Enterococcus hirae* increased significantly in the *LPK* group compared with the placebo group. Such compositional changes might play important roles in changes in lipid profiles.

Even though the abundance of *L. plantarum* was negatively correlated with abdominal fat area with borderline significance, no significant reduction in body fat mass was observed in the *LPK* group. There are several possible reasons for this. First, there might be a large variation in the amount of bioavailable *LPK*. Also, despite the significant changes of a few gut microbiota after *LPK* administration, their clinical effects might be small at very low levels. Second, the moderate baseline BMI of 27.3 kg/m^2^ of our study participants might not have been high enough to confirm the effect of *LPK*. Although the study group was allocated randomly, the uneven baseline levels of the markers of liver function and inflammation might have affected the study results. As our study was conducted in Korean population, further studies may help to generalize these effects to other ethnic populations.

## Conclusions

In this randomized, double-blind clinical trial, administration of *LPK* for 12 weeks did not lead to significant reductions in total fat mass or in body weight. However, significant decreases were found in total cholesterol and TG levels after *LPK* treatment. These data suggest that *LPK* might be a good auxiliary candidate as a microbiome-targeted therapy for treating dyslipidemia.

## Data Availability Statement

The data presented in the study are deposited in the Sequence Read Archive (SRA) repository, accession number (PRJNA777658).

## Ethics Statement

The studies involving human participants were reviewed and approved by Ethics Committee of Seoul National University Bundang Hospital. The patients/participants provided their written informed consent to participate in this study.

## Author Contributions

SL contributed to conception, acquisition, analysis, or interpretation of data, drafting the work or revising and final approval of the manuscript. MS and GN contributed to the acquisition, analysis, or interpretation of data, drafting the work or revising and Final approval of the manuscript. JC, HJ, and B-KK participated in the acquisition, analysis, or interpretation of data and final approval of the manuscript. All authors contributed to the article and approved the submitted version.

## Funding

This research was supported by the Korea Institute of Agriculture, Food and Rural Affairs (IPET) through the technology commercialization support project of the Ministry of Agriculture, Food and Rural Affairs (accession number: 818028).

## Conflict of Interest

JC, HJ, and BKK were employed by the company CKD BiO Corp.

The remaining authors declare that the research was conducted in the absence of any commercial or financial relationships that could be construed as a potential conflict of interest.

## Publisher’s Note

All claims expressed in this article are solely those of the authors and do not necessarily represent those of their affiliated organizations, or those of the publisher, the editors and the reviewers. Any product that may be evaluated in this article, or claim that may be made by its manufacturer, is not guaranteed or endorsed by the publisher.
