# Supplementary material for: Efficacy and Safety of Lactobacillus plantarum K50 on Lipids in Koreans With Obesity: A Randomized, Double-Blind Controlled Clinical Trial
Source: Front Endocrinol (Lausanne). 2022 Jan 19;12:790046. doi: 10.3389/fendo.2021.790046 (PMC8807682; doi:10.3389/fendo.2021.790046)

**Education for healthy lifestyle**

The participants were trained to follow the instructions given below for a healthy lifestyle from the run-in period to the end of the study. The other lifestyles that are not mentioned below are also instructed to maintain the usual habits. The instructions were developed based on the Dietary Reference Intakes for Koreans (KDRIs) published by the Ministry of Health and Welfare and The Korean Nutrition Society (http://kns.or.kr/English/Publication.asp).

1) Restrictions on the possible intake of foods with high fat (or high cholesterol) content

2) From the baseline visit, it is recommended that one exercise regularly

- exercise three times a week for 20 to 30 minutes a day

3) Prohibition on excessive consumption of major Korean food materials among the water-soluble dietary fiber feed foods

- consumption of less than 1/3 of mixed grains

- restriction on the consumption of barley, sweet potatoes, garlic, bean sprouts, onions, pears, etc.

4) Restrictions on excessive intake of fruits and vegetables

- fruits or fruit juice ≤2 times/day, vegetables ≤6 times/day

5) Prohibition on consuming concentrated pills of seaweed or vegetable

6) Prohibition on consuming vegetable extracts such as onion juice, garlic juice, black garlic juice, pumpkin juice, and fruits extracts such as pear juice and grape juice

7) Taking the drugs specified in the exclusion criteria is prohibited

- appetite modulator (suppressant/stimulant), diuretics, beta blockers, contraceptives, hormones (sex hormones, thyroid hormone, steroids), tricyclic antidepressants, lipid-lowering agents, anti-diabetic agents, laxatives, anti-inflammatory agents, etc.

8) Do not consume health functional foods that affect weight, lipids, blood sugar metabolism, and immune function

9) Prohibition on ingesting the foods (including health functional foods) that can affect intestinal health

- capsules containing probiotics (lactic acid bacteria) and prebiotics (such as food fibers, fructo-oligosaccharides), refined tablets, fermented oil, etc.

10) No alcohol consumption within 48 hours prior to the visit

11) Prohibition on excessive activities and restriction on exercise within 48 hours prior to the visit

12) No smoking on the day of the visit until the process is completed

**Supplementary Fig. S1**. Study design. DXA, dual-energy X-ray absorptiometry; CT, computed tomography.


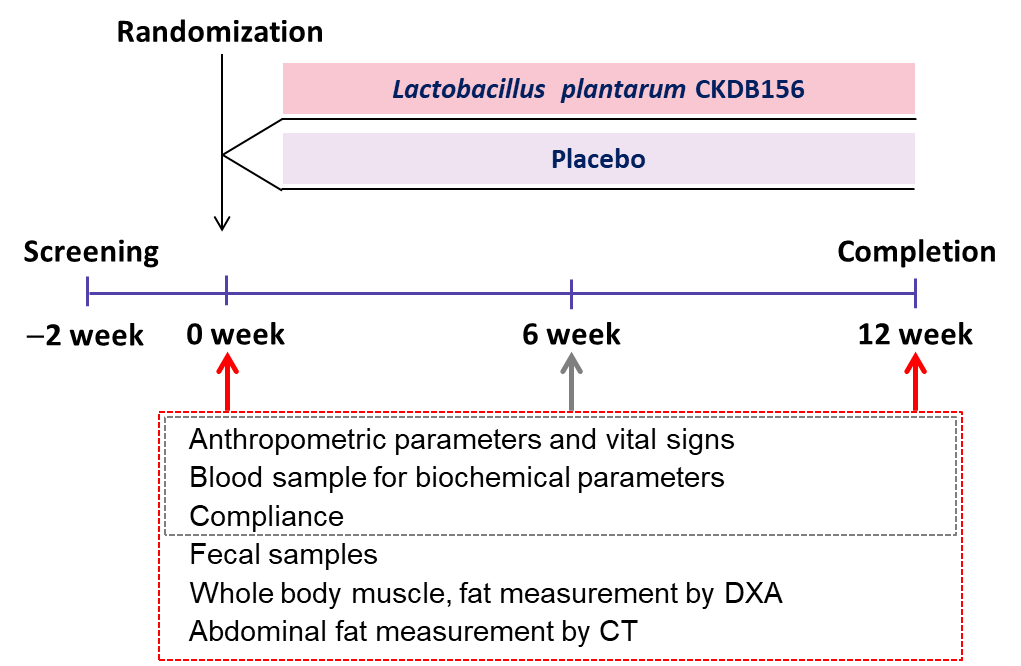


**Supplementary Fig. S2**. Correlation between clinical values and the abundance of microbiota, evaluated by Pearson’s method. VAT, visceral adipose tissue; SAT, subcutaneous adipose tissue; BMI, body mass index; TC, total cholesterol; TG, triglyceride; HDL-C, high-density lipoprotein cholesterol; LDL-C, low-density lipoprotein cholesterol. Each ‘p_,’ ‘g_,’ and ‘s_’ stands for taxa belonging to a particular phylum, genus, or species, respectively. ^*^P<0.05; [^§^](http://en.wikipedia.org/wiki/Section_sign)P<0.1.


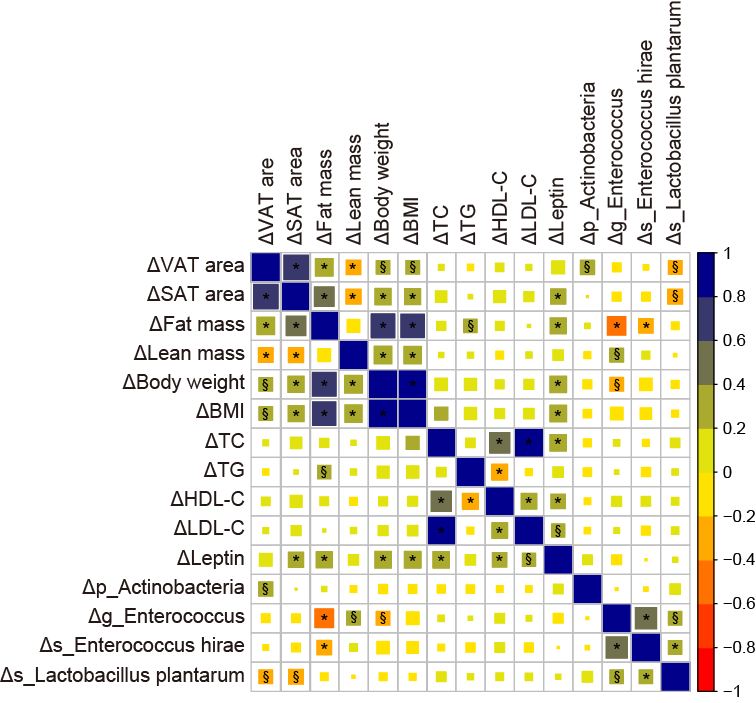

Supplement: Supplementary file 1 [file DataSheet_1.docx]
